# The “unknown territory” of goal-setting: Negotiating a novel interactional activity within primary care doctor-patient consultations for patients with multiple chronic conditions

**DOI:** 10.1016/j.socscimed.2020.113040

**Published:** 2020-07

**Authors:** Jamie Murdoch, Charlotte Salter, John Ford, Elizabeth Lenaghan, Alice Shiner, Nicholas Steel

**Affiliations:** a School of Health Sciences & Norwich Medical School, Faculty of Medicine and Health Sciences University of East Anglia, Norwich Research Park, Norwich, NR4 7TJ, UK; b Norwich Medical School, Faculty of Medicine and Health Sciences, University of East Anglia, Norwich Research Park, Norwich, NR4 7TJ, UK; c Institute of Public Health, University of Cambridge, Cambridge, CB2 0SR, UK

**Keywords:** UK, Goal-setting consultations, Primary care, Doctor-patient interactions, Conversation analysis

## Abstract

Goal-setting is widely recommended for supporting patients with multiple long-term conditions. It involves a proactive approach to a clinical consultation, requiring doctors and patients to work together to identify patient's priorities, values and desired outcomes as a basis for setting goals for the patient to work towards. Importantly it comprises a set of activities that, for many doctors and patients, represents a distinct departure from a conventional consultation, including goal elicitation, goal-setting and action planning. This indicates that goal-setting is an uncertain interactional space subject to inequalities in understanding and expectations about what type of conversation is taking place, the roles of patient and doctor, and how patient priorities may be configured as goals. Analysing such spaces therefore has the potential for revealing how the principles of goal-setting are realised in practice. In this paper, we draw on Goffman's concept of ‘frames’ to present an examination of how doctors' and patients' sense making of goal-setting was consequential for the interactions that followed. Informed by Interactional Sociolinguistics, we used conversation analysis methods to analyse 22 video-recorded goal-setting consultations with patients with multiple long-term conditions. Data were collected between 2016 and 2018 in three UK general practices as part of a feasibility study. We analysed verbal and non-verbal actions for evidence of GP and patient framings of consultation activities and how this was consequential for setting goals. We identified three interactional patterns: GPs checking and reframing patients' understanding of the goal-setting consultation, GPs actively aligning with patients' framing of their goal, and patients passively and actively resisting GP framing of the patient goals. These reframing practices provided “telling cases” of goal-setting interactions, where doctors and patients need to negotiate each other's perspectives but also conflicting discourses of patient-centredness, population-based evidence for treating different chronic illnesses and conventional doctor-patient relations.

## Highlights

• First study of GP goal-setting interactions for patients with multiple conditions.
• Goal-setting involves novel activities in an uncertain interactional space.
• GPs and patients may need to negotiate different framings of patient's priorities.
• GPs may actively align with patient's goal, patients may resist GP framing of goal.
• Negotiation of frames reveal conflicting discourses invoked at interactional level.

## 1 Introduction

Goal-setting, the sharing of realistic health and wellbeing goals by physicians and patients, is core to the theory and effective practice of personalised care planning and seen as particularly important for patients with multiple chronic and long-term health conditions. It involves “eliciting and clarifying patients' understanding of their condition, their values, outcome preferences and priorities … the choice of goals and priorities is not restricted to a prespecified list of professionally determined options” (Coulter et al., 2015). Rather than a focus on specific disease management strategies, a key principle of goal-setting is that the patient decides which goals they would like to achieve. Such a principle allows the possibility for patients to share concerns and priorities that, while related to their health problems, are grounded in the contextual realities of everyday life, otherwise understood as the patient's lifeworld (Barry et al., 2001). Goal-setting is therefore intended to create a space for the sharing of physical, practical, emotional, psychological and social concerns as they are oriented to and made salient by the patient. The doctor's role is to work collaboratively with the patient to identify how best to help them achieve their goals. This may include identifying medical interventions such as clinical tests and the management of medications, but may also involve forms of ‘social prescribing’ (Baddeley et al., 2016) such as referrals to support groups or practical suggestions for helping the patient carry out their daily activities. Indeed, the doctor may just need to ‘bear witness’ (Heath, 2012) to the patient's concerns without intervention.

The concept of goal-setting is therefore positioned firmly within a broader discursive framework of patient-centred medicine, shared-decision making and patient empowerment with a view to determining care for the patient that both respects them as individuals and might lead to improved health outcomes (Mead and Bower, 2000; Stewart, 2001; Michie et al., 2003; Epstein and Street, 2011). However, although goal-setting might appear to be a relatively straightforward process, it requires both doctor and patient to engage in a set of activities that represent a distinct departure from more standard consultations. While the detail of individual consultations obviously varies enormously, the conventional structure used in communication skills training (Silverman, Kurtz & Draper, 2013) and widely practised by doctors is one where the patient presents a problem and the doctor reactively recommends a course of action to address the problem, or to reduce the patient's risk of adverse outcomes from long-term conditions. This standard structure typically necessitates an interactional arrangement whereby the GP guides the sequence and transition across activities within the consultation. Goal-setting by contrast is a proactive approach, requiring doctors and patients to understand the patient's values, outcome preferences and priorities. The doctor is the expert on medical diagnosis and treatment, and has to acknowledge the patient's expertise in their desired outcomes, lifestyle, social and emotional context, and problem-solving strategies, all of which are central to goal-setting. Understanding how doctors and patients navigate goal-setting as a set of novel interactional activities therefore requires in-depth investigation if such an approach is to be widely recommended and implemented within healthcare settings.

The challenges of goal-setting as an interactional task have already been identified in observations and interviews with staff and patients in neurological and stroke rehabilitation settings (Barnard et al., 2010; Levack et al., 2011; Plant et al., 2016; Keel and Schoeb, 2017), physiotherapy treatment sessions (Parry, 2004; Schoeb et al., 2014), and between a range of healthcare professionals, (including doctors) and patients with chronic conditions (Franklin et al., 2019a). This work has identified that patients do not necessarily have clearly defined goals for themselves, and that clinicians can be seen to dominate goal-setting activities to ensure they are clinically appropriate and achievable, but despite this, patients may resist professionals’ attempts to control which goals are set.

Such potential difficulties in how doctors and patients conduct goal-setting consultations have clear implications for supporting people with chronic conditions, as well as for ongoing debates about the distribution of power and the function of asymmetrical interactional structures for realising patient-centred consultations (Pilnick and Dingwall, 2011). Goal-setting is linked to self-management of chronic conditions over time, managing risk and lifestyle choices (Coulter, 2015). However, understanding that patients' priorities do not always match this biomedical agenda (Schoeb et al., 2014) brings wider tensions between clinical and patient-centred policies into sharp focus at an interactional level. If goals are to be patient-centred, with GPs facilitating that process, then goal-setting relies on both GP and patient having a mutual understanding of what the consultation is about, the principles that underpin it, the type of conversation that will take place and what the purpose is of each activity within that process. In addition, both parties need to negotiate potentially conflicting agendas about what they want out of the consultation and crucially if and how they view the particular individual's priorities as goals.

Goal-setting for patients with multiple long-term conditions arguably creates an additional interactional challenge, with goals to improve living with one condition potentially compromising clinical requirements to manage other conditions effectively. Furthermore, doctors and patients may differentially conceptualise what it means to ‘live well’ with different chronic conditions (Morgan et al., 2017). There is no previous research that has specifically examined how goal-setting is interactionally achieved for patients with multiple chronic conditions within primary care doctor-patient consultations. Instead, studies have predominantly focused on measuring the impact of goal-setting on different behavioural outcomes such as activity levels or self-management plans (Bodenheimer and Handley, 2009). Whilst there is some qualitative interview-based evidence that doctors view goal-setting as an integral component of shared decision making with patients with multiple long-term conditions (Vermunt et al., 2019), there is limited understanding of how such views are translated into clinical consultations to create goals that are meaningful to patients. A recent qualitative study adopted Bourdieu's concept of habitus to illustrate how patients' expressions of goals within research interviews were temporally and structurally situated, emphasising the importance of considering how social positioning impacts on individual's investment in self-management (Franklin et al., 2019b). The findings reported here offer an extension of this insight into the doctor-patient encounter, using data collected as part of a larger cluster-randomised feasibility study (Ford et al., 2019). We have already reported findings from a thematic analysis of the video-recorded doctor-patient consultations and focus group data collected in this study, which identified that the core components of the goal-setting process included prior preparation for goal-setting for patients and GPs, collaborative goal-setting, and GPs legitimising or “bearing witness” to patients' goals (Salter et al., 2019). However, we also identified a number of challenges for GPs and patients in navigating goal-setting consultations. Doctors reported being in “unknown territory” as they struggled to deliver consultations driven by patients' goals, and patients expressed puzzlement at how the consultation successfully enabled them to achieve their goals. These findings suggest that goal-setting was difficult to accomplish within an institution that has historically cast GPs as expert decision-makers and active agents solving patient problems, adjudicators and gatekeepers of patient care, rather than necessarily legitimising patients' priorities and collaboratively figuring out strategies for achieving goals.

### 1.1 Theorising goal-setting consultations as novel interactional activities

Goffman, in his observations of everyday interactions, highlighted how social activity, comprising verbal and non-verbal actions, is organised by the normative expectations participants bring to bear on the activity taking place, how they make sense of the situation to answer the question “what it is that is going on here” (Goffman, 1974). This psychological sense-making of face-to-face interactions is what Goffman referred to as the interactant's “frames” of the norms, roles and communicative expectations of the interaction. Importantly, Goffman identified evidence of how frames can be switched, treated as “out-of-frame,” or disattended to by other interactants in uncertain interactional spaces (1974:10). Talk and non-verbal behaviour therefore has the potential to reveal such moves, providing manifestations of alignments, shifts or resistance to different frames as interactions unfold. Interactional Sociolinguistics, principally accredited to the work of John Gumperz (Gumperz and Hymes, 1972), has been hugely influential in making such connections, setting out a general theory of verbal communication that draws both on Goffman and use of conversation analysis (Sacks et al., 1974) to:

*“take the speech event as the unit of analysis rather than community-wide linguistic and cultural norms, to see that culture did not stand outside talk but was constituted in and through situated speaking practices”* (Gumperz, 1982)

Goffman's frames provide an interpretive concept for empirically connecting small-scale interactions with wider social forces and have already been applied to make sense of clinical interactions, (Tannen and Wallat, 1987; Coupland et al., 1994; Roberts et al., 2004), highlighting how doctor and patient expectations and understandings about what doctors and patients do within clinical consultations shapes their framings of the activities and ongoing talk.

Goffman's concept of frames is particularly pertinent within goal-setting consultations, which require a different genre of communication from that used in a typical GP consultation, representing an intervention that “disrupts” (Hawe et al., 2009) a complex system of historically institutionalised expectations, discourses and practices. Patients and GPs will therefore draw on these historical reference points in framing goal-setting interactions which will subsequently shape how both parties use the resources (i.e. knowledge and skills) available to them and consider appropriate to deploy within the activities of the consultation. The embodied experience of the patient (i.e. their physical, social and psychological circumstances), the history of their interactions and the respective GP and patient agendas will inevitably intersect with such framings of the different activities. This conceptualisation of frames is therefore not one with fixed psychological properties, but is open and fluid, subject to negotiation and change. It also raises the possibility of interactional inequalities in the distribution of the different meanings of goal-setting within the doctor-patient encounter, and the activities within it, which are likely to be consequential for how the GP-patient interactions proceed. For example, Roberts et al. (2004) highlighted how doctor-patient consultations become protracted and subject to misunderstandings when patients have not been socialised to consultation conventions. Gumperz (1999) referred to this as “mini-tragedies” of interactions, whereby a lack of shared understanding has potentially important material consequences for what follows, in this case, how patients are treated and cared for.

To contribute to theoretical understanding of goal-setting interactions, we investigated how Goffman's notion of frames helps to understand how the broader policy agendas of chronic illness management and goal-setting are recontextualised within the interactional activities of preparation, eliciting and setting of patients' goals, action planning and review. Such understanding is essential for considering the achievements of goal-setting as an intervention, for training GPs, for supporting patients to action their own health and wellbeing goals, and for critically evaluating how goal-setting as an intervention might be optimally delivered within general practice or other healthcare settings.

## 2 Method

We analysed video recorded goal-setting consultations from the intervention arm of a cluster-randomised controlled feasibility trial of goal-setting, full details of the study have been reported elsewhere (Ford et al., 2019; Salter et al., 2019). The study took place between November 2016 and July 2018 in Norfolk and Suffolk, UK. It was set in six general practices and recruited patients considered by their surgery to be in the top 2% most at risk of hospital admission, eligible for a new care plan, living with more than one long-term condition and able to communicate verbally. Patients were excluded if their GP considered them unsuitable to participate in a goal-setting consultation, for example if they had advanced dementia or acute psychosis. Research ethics approval was obtained from the NHS Research Ethics Committee (16/EM/0411).

The goal-setting intervention involved GPs in a brief 3 h training workshop using a structured patient-centred stepped approach based on established models of communication and shared decision making (Silverman, Kurtz & Draper, 2013; Elwyn et al., 2017) and informed by S.M.A.R.T goals (Specific, Measurable, Attainable, Realistic and Time bound). See Salter et al. (2019) for more details. Patients were provided with a 3-page A4 goal-setting sheet prior to the first appointment with room for note making and three trigger questions including: what are your goals, why are these goals important to you and, what are the first steps you would like to take towards achieving this goal or goals? Patients were asked to set up to three goals and to bring the goal-setting sheet to the first appointment with a participating GP. GPs documented the agreed goals and provided support to help patients achieve their goals. Control practice patients received usual care.

In this analysis, we focus on the data collected from the three practices randomised to deliver a goal-setting intervention. Intervention participants comprised a total of 5 GPs and 22 patients with an average of 5 chronic conditions per patient (see Table 1). The goal-setting consultations were 20 min in duration and video-recorded following written consent from participating patients and GPs. Recordings were transcribed verbatim, including non-verbal actions, based on Jefferson's transcription conventions (Jefferson, 2004). One patient consented to audio-recording only. During transcription, all identifying features were removed or replaced with pseudonyms. Recordings amounting to a total of 673 min were initially watched independently and then by the whole study team comprising the study researcher, a GP and two social scientists with expertise in communication skills. Emerging results were discussed over a six month period. Analysis began by describing the gross structure of whole transcripts to delineate activity types (Murdoch et al., 2015). We sought deviant cases to explore the complexity of the task and the communicative challenges posed.

**Table 1:** Characteristics of practices randomised to goal-setting intervention, and of participating GPs in those practices.

Practice characteristics	Practice 1	Practice 2	Practice 3
Practice rurality a	Village - less sparse	Town and fringe - sparse	Town and fringe - sparse
Practice population, range, *n*	5000 to 9900	10000 to 14900	5000 to 9900
IMD decile	7	5	7
**Characteristics of participating GPs**			
Male sex, n	2	1	1
Female sex, n	0	1	0
Employment status	Partners, 2 PT	Partners, 2 FT	Partner, PT
Time qualified, years	GP014, >20 GP018, 10 to 20	GP025, <10 GP026, 10 to 20	GP038, 10 to 20
**Characteristics of participating patients**			
Male sex, n	3	4	3
Female sex, n	7	4	1
Average number of conditions^b^	4.7	4.9	6.5

a Office for National Statistics indicator 2011. IMD = Index of Multiple Deprivation (1 = most deprived and 10 = least deprived). Partner = GP with responsibility for the practice. PT = part time. FT = full time. b Based on Barnett list (Barnett et al., 2017).

The subsequent activity-based analysis of the video recordings explored how GPs and patients enacted key aspects of the goal-setting consultations including preparation and opening, eliciting goals, assessing options, making goals S.M.A.R.T, decision making, summary and evaluation and closure (Salter et al., 2019). A central focus was on patterns of interaction within each activity (Gumperz, 1982), searching for evidence of how GP and patient framed each activity and how frames were negotiated as interactions proceeded. This required treating the talk and non-verbal actions of doctor and patient as evidence of participant frames, with a consideration of how negotiation of frames revealed a wider distal context which needed to be negotiated in the proximal context of goal-setting interactions. The use of conversation analysis was adopted to examine how the articulation of respective framings was consequential for the subsequent discussion and to identify evidence of how frames were negotiated and shifted as discussions around goal-setting evolved. This evidence was sought in how each speaker oriented to the previous turn, how doctor and patient could be seen to attempt to re-frame the other's understanding, and how goals and patients' priorities came to be defined and categorised. This analytical work enabled us to reach an understanding of the extent to which the goal-setting tasks were mutually shared, driven by one party, contested, and how such communication challenges were managed (Heritage and Maynard, 2006). In doing so, we were able to generate theoretical generalisations of how GP-patient goal-setting consultations are likely to be socially structured and identify how goal-setting might be successfully achieved.

## 3 Findings

In this section we present three broad patterns that we identified in how GPs and patients enacted goal-setting discussions, including GPs checking and reframing patients' understanding of the goal-setting consultation, GPs actively aligning with the patient's framing of their goal, and patients passively and actively resisting GP attempts to reframe patients' priorities into measureable goals. Our examples provide “telling cases” (Mitchell, 1984) of the relationship between goal-setting as an intervention and the primary care context in which it was introduced. In each case, we present the transcript using Jeffersonian transcription conventions (see Box 1). In all but the first extract we have also included the GP's or the patient's bodily conduct to provide further evidence of our interpretation of the talk taking place.

Box 1 **Transcription conventions (Jefferson, 2004)**

**Table:** 

(0.4)	A silence, measured in tenths of a second
(.)	A micropause, hearable but too short to measure.
>he said<	‘greater than’ and ‘lesser than’ signs enclose speeded-up talk. Occasionally they are used the other way round for slower talk.
Underlining	indicates emphasis; the extent of underlining within individual words locates emphasis and also indicates how heavy it is.
↑ ↓	Vertical arrows precede marked pitch movement, over and above normal rhythms of speech. They are used for notable changes in pitch beyond those represented by stops, commas and question marks.
she wa:nted	Colons show degrees of elongation of the prior sound; the more colons, the more elongation.
[ ]	Square brackets mark the start and end of overlapping speech. They are aligned to mark the precise position of overlap as in the example below.
^°^↑I know it,^°^	‘degree’ signs enclose hearably quieter speech.
.hhh	Inspiration (in-breaths); proportionally as for colons.
£yes£	Smile voice
#sad#	Talk between markers is croaky
(?)	Unclear talk
?	Rising intonation

Alt-text: Box 1

### 3.1 Framing the GP-patient consultation as a goal-setting discussion

When patients arrived for their goal-setting consultation, they were asked to bring their prepared goals and associated paperwork with them to discuss with the GP. How patients understood and prepared for this conversation was therefore important for how the interaction commenced. Where patients arrived at the consultation with an understanding of goal-setting that matched the GP's understanding, the discussion could be seen to move smoothly on to eliciting the goals the patient might have, for example:

**Table:** 

	GP or Patient (Pt)	Words spoken/sounds
1 2	GP014	u::m, (0.6) have you thought of any goa:::ls that you: >that we could< (.) maybe (.) discuss and refi:ne
3 4 5	Pt 111	↑ye:::s (0.4) e::rm, (0.4) one thing was I wanted to keep myself (0.4) u::::m, (0.6) fi::t a:nd, (0.6) try and get more healthy, (.) and mobi:le as much as possible really,
6	GP014	↑ye:s
7 8 9	Pt 111	(°for°) my main ai::ms, (0.6) co::s I love doing my gardening I want to be able to do:, (0.4).hhh obviously there's a lot what I can't do but, (0.4) I want to be able to d-, (0.4) carry on doing the bits I ca:n
10	GP014	↑ye:s

#### 3.1.1 Extract 1: patient arrives at consultation with prepared goals

In this extract, both GP and patient offer a variation on Heritage and Sefi's classification of marked acknowledgement tokens (Heritage and Sefi, 1992, p391-402), with “yes” delivered with a notable change to a higher pitch and an elongated vowel (lines 3, 6, 10). This type of acknowledgement functions to indicate that the listener has heard the information being provided in the previous turn (Silverman, 1997, p.129–30), and that it makes sense with their own framing of the task at hand; seen firstly with the patient's response to the GP's elicitation of goals, and then with the GP who indicates support as the patient elaborates on his desire to keep fit. However, some participants did not display a clear understanding of the aim of the consultation and did not arrive with pre-specified goals. This led to the GP explaining the purpose and then beginning a process of searching for problems, concerns and goals the person might have. In these instances, the analytical focus was immediately set in epistemic terms, with the reasons for holding the consultation, the principles of personalised care, the aims and agenda of goal-setting, and the genre of communication within it needing to be communicated to the patient before the consultation could proceed. GPs could be seen to attempt to reframe patients' understanding by informing them that the consultation was: “*all about you*”, understanding “*what is close to your heart*” (GP038 in consultation with Pt 304), or more specifically, “i*t's to look at ways to help your ongoing medical problems … by the way of setting goals*.” (GP026 in consultation with Pt 203).

Almost inevitably, where patients were uncertain about the reason for the consultation, reaching a point of clarity regarding what goals were important to the patient was difficult to achieve, relying on patients being able to articulate at that moment what mattered to them. In addition, the GP needed to be alert and sensitive about which areas to follow up further. In Extract Two, a patient arrives without prepared goals and apparently unsure about the aims of the research, stating “it's just to help old people isn't it?” After attempting to reframe the patient's understanding, the GP then asks a focused question aimed at specifically eliciting the patient's goal.

**Table:** 

	GP or Patient (Pt)	Words spoken/sounds	Bodily conduct
1 2	GP025	so: (0.4) do you have any goa::::ls or targe::ts that (.) you're <aimi::ng fo::r?>	
3 4	Pt 205	just to keep well ha ha [ha ha ha ha huh huh]	
5 6	GP025	[↑yea::::h (.) yeah I] think that's a £very good o[:ne actually, huh] I think£.hhh	
7	Pt 205	[ha ha ha ha ha ha]	
8	GP025	>keeping well< and keeping happy::	
9	Pt 205	yeah [yeah fine (.) yeah,]	
10 11	GP025	[certainly (.) ye:s] it's a really good starting point >now look,<.hhh ↑let's tea::se that out a little bi:t	GP does hand rolling motion to emphasise teasing out
12	Pt 205	Right	
13	GP025	have you got any proble::ms at the momen**t**?	
14		(2.0)	Pt has mouth wide open as if lost for words
15 16	Pt 205	HHHHHHH (0.4) u::m, (0.6) I don't think so, my husba:nd is my problem, (0.6) [because] he's got =	
17	GP025	[↑mm::::]	
18 19	Pt 205	= a bi:t o:f (.) dementia >so have I:< (0.4) but we:: (0.6) have lots of thi:ngs (.) like that so I'm thinking for hi::m as well as me	
20	GP025	↑ye:s	

#### 3.1.2 Extract 2: patient arrives without prepared goals

The patient's initial response is provided in broad terms “just to keep well” (line 3) which for the purpose of goal-setting requires greater specification which the GP attempts to achieve, *”Have you got any problems at the moment*?” (line 13). However, for the patient, this question seems to be unexpected, at line 14 she can be seen staring at the GP with an open mouth as if lost for words. After a 2 s pause the patient reports that both she and her husband have dementia. However, the patient's talk does not overtly express dementia as a problem that she clearly wants the GP to follow up on and configure into some kind of goal. The patient's statement is prefaced with a mitigating phrase, “Um. I don't think so” (line 15), she positions her husband's dementia as “my problem” (lines 15–16) and the disclosure of her dementia, “>so have I:<”, (line 18), is delivered at a higher tempo to the surrounding talk as if this is just incidental information. The significance of dementia in her life is then minimised, “but we have lots of things like that,” (lines 18–19) as just one part of a number of everyday problems that she needs to manage. With the patient's response delivered in this way, the GP may have easily missed a potential opening to pursue problems of dementia or being a carer, or was faced with an interactional dilemma in how to respond appropriately. Instead a general chat follows, including details of the person's employment history and the daily routine of the patient and her husband who she cares for. Ultimately, a goal is set for the diabetic nurse to contact the patient to discuss the patient's insulin dose.

### 3.2 GP actively aligning with patient's framing of goal

Once the GP had elicited the patient's understanding of the consultation, and as we frequently observed worked to align the patient's framing with their own, then the key activity of eliciting the patient's goals could begin. As we have previously reported, (Salter et al., 2019), asking patients what mattered to them seemed to have a powerful legitimising effect on which problems and goals were presented during the consultation, in some cases allowing problems to be aired that had not previously been disclosed. However, how the GP then responded to the problems or goals was often dependent on which goals patients presented and the variety of interactions that followed revealed different components of what active collaboration entailed. In Extract 3, the patient arrived at the consultation with two specific goals, gaining weight and stopping smoking, both of which align easily to a concrete medical intervention. The task of setting a measurable goal is also relatively straightforward, reducing or stopping smoking is easy to measure in quantifiable terms. In this case, the interaction progresses smoothly as the GP and patient work to agree on realistic targets and mechanisms for achieving the goal. Interestingly, it is the patient who is adamant that the goal is to stop smoking. Initially holding back from firmly agreeing with the GP's suggestion to attend a stop-smoking clinic (mm:) at line 4, the patient interrupts at line 7 with a clear statement that “No I want the goal to be stop smoking.” It is the GP, rather than the patient, who actively aligns and then supports the patient's framing of her goal, offering a justification for her decision “because we've got six months”.

**Table:** 

	GP or Patient (Pt)	Words spoken/sounds	Bodily conduct
1 2 3	GP026	oka:y (.) so::: (1.0) well the sma:rt goal is I think, (0.4) we want to:: (.) not beat about the bush we could say the goal could be just attendi:ng the (.) the ↑smoking clinic	GP writes on paperwork and then looks up at Pt and uses open rolling arm motion to invite Pt's agreement
4	Pt 206	mm::,	Pt keeps eye contact and nods slightly
5 6	GP026	and we could set that as the goa::l, th[at's a simpler one]	GP points to what he has just written
7	Pt 206	[↑no::: I want the] goa:l,	Pt nods to emphasise her statement
8	GP026	>to be< stop smoking	
9	Pt 206	stop smoking	Pt uses sharp downward arm and hand movement to emphasise the finality of the ‘stop’
10	GP026	because we've got 6 mo:nths,	GP goes back to writing on paperwork
11	Pt 206	°yea:[:h°]	
12 13	GP026	[so] I think that's reasonable [stop] smoking =	GP continues writing (this target) on paperwork as he speaks
14	Pt 206	[°mm::°]	
15	GP026	= .hh within s- (0.6) within (0.4) the six months	GP continues writing (this target) on paperwork as he speaks
16	Pt 206	yea:h	

#### 3.2.1 Extract 3: GP aligning with goal set by patient

However, reaching a point where GPs could be seen to collaborate with patients was not always a straightforward process. In Extract 4, the patient has arrived at the consultation with three clearly defined goals - reduce medication, increase exercise and lose weight. In contrast to Extract 3, the patient seemed to lead the GP throughout the consultation, with the GP appearing to take a while to align his position alongside the patient. At line 6, the patient recalls a previous encounter with a rheumatology consultant where he attempted to express his concern that his arthritic medications were providing little benefit and his desire to see how he reacted without them.

**Table:** 

	GP or Pt	Words spoken/sounds	Bodily conduct
1 2	GP018	u:::m, >is there< anythi:ng in particular >you wanna< a:sk befo:re we get underway with the: (0.4) nitty gritty of it?	GP looks and points at paperwork then turns to Pt smiling
3 4 5	Pt 109	.hh we::ll, (0.4) ↑not really I mean e:::r (0.4) obviously, (0.4) one of my priorities would be if I cou:ld, (0.6) possibly cut back on some of the ta:blets,	Pt shifts to take up GP's eye contact
		**[[17 lines omitted, approx. 21 secs elapsed, patient specifies medication for arthritis]]**	
6 7	Pt 109	and u:::m, (0.8) but as I tried to explain to the lady at the hospital she: w- you must keep taking them every da::y	Pt cups hand over edge of desk for emphasis
8	GP018	mm, mm::	
9 10	Pt 109	>I said well< the only thing i:s, (0.4) it's the same as you get headache tablets and tha:t,	
11	GP018	mm (.) [mm,]	
12 13	Pt 109	[you] do::n't (0.6) prolo:ng keep taking them >because,< (0.4) you don't know whether you're getting any bette::r >or anything<	
14	GP018	mm:: (.) [mm]	
15 16	Pt 109	[be ]cause they're supressi:ng how you, (0.4) how you really a::re.	Pt uses hand gesture for emphasis
17 18	GP018	mm: and u::m, (0.4) let me just (.) >↑do you mind< ↑if I get get your lette:r from the: u:::m, [rheumatologist doct]or °just have a°	GP turns back to computer screen and moves mouse
20	Pt 109	[↑no not at all sir ]	
21	GP018	°↑no°	
		[[**180 lines omitted, approx. 8 min elapsed**. **GP reviews all the patient's medication as well as discussing other goals]]**	
22 23 24	GP018	so it looks li:ke (0.4) in terms of trying to reduce the tablets down a bi:t (.) u:::m, (0.6) certainly (0.4) the::: >rheumatoid ones I< (.) I think you're ri:ght, a::re, (0.4) o:nes that we could focus on,	GP is looking a screen and points towards it with index finger
25	Pt 109	mm::	

#### 3.2.2 Extract 4: GP negotiating medical authority with patient goal

During the patient's anecdote about the consultant, the GP shows signs that he is acknowledging the information provided by the patient “mm” without offering a clear indication that he is currently aligning with the patient's position (lines 8, 11, 14). As the interaction proceeds, it becomes clear that this reticence from the GP is initially based on a need to acquire more information regarding which medications the patient takes. However, rather than then pressing forward with actions to help the patient achieve his goal, the GP then asks the patient if he minds (lines 17–18) if he retrieves and reads the letter from the rheumatologist. This particular feature arguably offers something distinct about how GPs may implicitly frame and enact goal-setting consultations compared with conventional doctor-patient consultations. Instead of just opening the letter, the request seems to hint at two unspoken principles that the GP needed to negotiate. Firstly, that setting goals is not necessarily a linear process whereby the GP simply follows the patient's agenda. Rather it is still contingent on how this agenda aligns with an authoritative medical view, in this case an absent third party (the consultant) and the GP's own clinical judgement. Secondly, that because the goal-setting conversation has been framed as patient-led, the GP activates a particular rule of engagement within this interaction, that if the GP wishes to undertake any activity that has the potential to derail the patient's agenda then the GP must ask the patient's permission. Here we see the intersection of these two principles firstly in how the GP asks the patient's permission and then at line 20 with the patient who upholds the GP's position as authoritative and as an agent of change, “no not at all sir.” While the GP does eventually align with the patient's wish to reduce his medication, it is only after lengthy interactional work which involves determining if the patient's goal is acceptable within a medical framework, shown by the GP's statement at lines 22–24 that “it looks like … I think you're right”.

### 3.3 Passive and active resistance to GP framing of goal

In contrast to consultations where patients and GPs reached a point of agreement and were able to progress with setting goals, we identified a number of instances where GPs did not easily align with patient's priorities and patients resisted how GPs framed their goal. In Extract 5 the patient has completed the goal-setting paperwork prior to the consultation, specifying that he would like to reduce the medication he takes, return to his previous level of wellbeing before he had a bout of pneumonia, commence social activities and physical work, and to stabilise his atrial fibrillation and diabetes. The patient also reports that he has already reduced his medication following a recent consultation with another doctor. The patient then reports that he does not take any medication for diabetes and he has not altered his diet to manage condition. A difficult interaction follows with the GP asserting his view that the patient's diabetes needs to be managed with medication and set as a goal.

**Table:** 

	GP or Pt	Words spoken/sounds	Bodily conduct
1	GP026	well I [think, (.) >you know< y]ou you're raising =	
2	Pt 202	[°huh huh huh huh huh huh°]	Pt nods faintly/slightly, blinks rapidly
3 4	GP026	= this question of diabetes and >as you've been< [talk]ing about it I've just been having quick =	Pt remains still
5	Pt 202	[°mm:°]	
6 7 8 9	GP026	= glances at your notes and,.hhh you certainly ↑do: have diabetes (0.4) and >it is< ↑cer- >and ↑that and that< ↑certainly this diabetes of older a::ge (.) and it's (.) °t-° gradually deteriorating >it's getting< a little worse it's no: it's not in a dra↑matic state	GP uses hand and arm movement to indicate an upward trajectory
10	Pt 202	°↑yea::h°	Pt nods gently then remains still
11 12 13 14 15 16 17	GP026	but it nee::ds closer management fro::m, (.) from a ↑doctor's point of view it needs closer management it's not, (0.4) e:::r under contro::l, (0.4) it's not ↑wildly ↑out of control but it's creeping that way (.) u::m (.) a::nd you are probably a patient that should be on medication (.) for diabetes and you're not at the moment, (0.4) um and I think this is perhaps something that would be a good health goa::l to look a:t	GP places right hand on heart At ‘creeping’ GP repeats hand movement in upward trajectory
18	Pt 202	°↑mm mm°	
19 20 21 22 23 24	GP026	um to keep this under contro:l to prevent, (0.4) admissions to hospital (0.4) °a:::h° so on and so fo:rth so I think one goal could certainly be around (0.4).hhh diabetes, one could certainly be around, (0.4) medication and explaining medication and one could certainly be about trying to, (0.4) e:::r recover (0.6) °e::r° following the recent illnesses,	Pt looks down at paperwork on desk. GP makes repeated pointed gesture at paperwork.
25	Pt 202	°hm ↑mm°	
26	GP026	°e::r° I think that's everything you've writte::n,	
27	Pt 202	that's right [the:re]	
28	GP026	[do::wn]	
29	Pt 202	ye::s	
30	GP026	↑yep,	

#### 3.3.1 Extract 5: passive resistance to GP's framing of patient goal

At this point in the consultation there is no explicit discussion of the tension between effectively managing diabetes and meeting the patient's stated goal to reduce medication. However, in contrast to Extract 1, the GP's assessment and treatment recommendation is met with unmarked acknowledgements, “mm” or “yeah” delivered as softer speech (lines 5, 10, 18, 25). In a study of patient agency and treatment decisions, Koenig (2011) found that such unmarked acknowledgements indicate patients passively resisting GP recommendations, which GPs typically follow by extending their treatment recommendation in pursuit of patient acceptance as the normative response. Here we see such minimal contributions by the patient followed by extensive GP turns where he presents the “doctor's point of view” (lines 11–12) and asserts that “one goal could certainly be around diabetes” (lines 20–21). However, instead of directly addressing the tension between his recommendation and the patient's wish to reduce medication, the doctor attempts to change the patient's framing of their goal from reducing medication to “explaining medication” (line 22). Following this extract, the consultation then switches to another goal, to increase the patient's physical activity, which again involves a difficult interaction as the patient resists the GP's attempt to set a “progressive walking programme” as a goal, by reclassifying it as “well walking programme, I think we will leave it at that to start with.” The discussion then returns to diabetes and in Extract 6 we can see the tension re-emerge between the patient's goal to reduce medication and the GP's agenda to manage the patient's diabetes more effectively.

**Table:** 

		Words spoken/sounds	Bodily conduct
1 2 3 4 5 6	GP026	>and then the< ↑thi::rd one is definitely this diabetes one I think (0.4) I don't know whether you agree: it's >one of< you:r (0.6) highlights was about, (.) making sure that your >comorbidities< are well managed (.) >y- y-< diabetes being one of them (0.4) I ↑certainly think there is, (0.4) room for you to be on a diabetes medication so (0.4) u::m,	GP turns over paper work he has been filling in and points to patient's paperwork. Pt glances down at paperwork which remains upside down to Pt.
7	Pt 202	which I would, (1.0) not (.) wish fo:r	
8	GP026	no[:]	
9 10	Pt 202	[i]f at all possible (0.6).hh I don't want to add, (0.6) pi:::lls or thi:ngs (0.6) other than the:: (0.4) check the regular (0.4) checks	
11		(0.8)	
12 13 14	GP026	°e:::r° yes and we ma::y, (0.4) >there are mayb-< there are many options for managing these thi:ngs (.) some are diet related some are pill related	GP opens hands palms facing upwards to indicate weighing up options
15	Pt 202	mm[::]	
16 17	GP026	[we] may not be able to:: a °ch-° you kno:w (.) say well we ↑can't use medication,	
18	Pt 202	mm	
19 20 21	GP026	because, (.) one of your other stated goals is to (.) keep enjoying what's left of (.) l- l:ife and [to be healthy and] we may need a medication to=	
22	Pt 202	[(?) (.) mm:]	
23 24 25	GP026	= prevent the diabete::s, (0.4) limiting life. (0.6) and I think >you're you're you're nodding awa:y< and I think that's (0.4) you know (0.4) [we need to] have =	GP holds both hands out and leans forward for emphasis when saying “limiting life”
26	Pt 202	[↑mm: (.) mm]	

#### 3.3.2 Extract 6: from active to passive resistance to GP goal-setting

Stivers defines active resistance as “an action that implicitly or explicitly questions or challenges the physician's treatment recommendation, including proposals or alternative treatments, (Stivers, 2005, p52). In contrast to the muted acknowledgements seen in Extract 5, we see the patient displaying active resistance in these terms, explicitly articulating his reluctance to take further medication (lines 7, 9–10). This seems to create a dilemma for the GP whose response reveals tensions between two incompatible ways of framing the consultation, between one view of goal-setting as being patient-led and another which is underpinned by clinical evidence that is translated into decisions by GPs. The GP seems to be fully aware of this dilemma, verbalising his own internal struggle to identify the correct way forward, *“we can't use medication because one of your other stated goals …”,* (lines 16–19), *“And we may need a medication to prevent the diabetes limiting life”* (lines 21–23)*.* Apparently to help resolve his problem, the GP looks to the patient for assistance, *“you're nodding away”* (line 24). However, as the GP talks the patient once again provides unmarked acknowledgements (lines 15, 18, 22, 26) suggesting his own retreat from active to passive resistance. Unable to reach a resolution the GP ultimately suggests a referral to a diabetes nurse, which functions to substitute a mechanism of action for the patient's goal.

## 4 Discussion

We have shown that when GPs and patients attempt to set goals together they may need to reorientate their respective framings of how the consultation will proceed. This reorientation demands a shift away from insititutionalised conventions and expectations about the types of activities that occur in GP consultations, to an unknown territory where the rules of engagement may be uncertain. For this reason, goal-setting consultations are vulnerable to mismatches in understanding about “what is going on here?”, in terms of what goal-setting is about, what counts as a legitimate goal, if and how goals should be measured and over what time period but also in terms of what types of GP and patient actions are legitimate within each activity.

These findings have clear links with previous research that has already emphasised the difficulties of goal-setting in other healthcare settings. Schoeb et al. (2014) identified the difficulty patients receiving physiotherapy had in formulating goals. Clinical rehabilitation teams have also been observed reformulating patients' wishes into goals that were considered acceptable and achievable, as well as identifying patients' strategies to resist these attempts through their own reformulations (Barnard et al., 2010). Similarly, Levack et al. (2011) and Franklin (2019a) found clinically recommended goals being privileged over patients’ goals, thereby undermining the ethos of patient-centredness in these consultations.

Our research extends these findings to primary care doctor consultations with patients with multiple chronic conditions, and considered by their surgery to be in the top 2% most at risk of hospital admission. Our data provide “telling cases” (Mitchell, 1984) of wider discursive tensions between biomedical perspectives of effective chronic illness management and prevention, conventions of doctor-patient relations and the idea of goal-setting as being patient-led. As a consequence of these broader tensions, GPs and patients could be seen to engage in “re-framing” communicative practices as they attempted to make sense of goal-setting activities and how goals should be determined, defined and categorised. This included explanations of goal-setting as being “all about you”, reconfiguring priorities as goals, reclassifying goals, (e.g. from “progressive walking programme” to “walking programme”), substituting mechanisms of action for goals, and negotiation of uncertain rules of engagement. Such practices illustrate doctors and patients negotiating not only the other's perspective but an uncertain discursive space where competing discourses of healthcare are being invoked at the point of delivery.

Our data showed that under these circumstances, the principles of goal-setting as being patient-led, patient-centred and involving GPs actively collaborating with patients to achieve their goal, were sometimes difficult for GPs to put into practice. Where patients did not have a clear understanding of what the consultation was about GPs actively attempted to reorientate the patient's framing to align with their own, leading to extensive searching for goals that might be relevant to the patient and ones the GP was willing and able to support.

Patients sometimes presented with goals (e.g. stop smoking) that GPs could immediately buy into and work with the patient to figure out a strategy. Here the notion of goals as being patient-led manifested through the interactional sequences with patients clearly asserting their goal and the GP showing alignment to the patient's position. In such cases, patients may have been well attuned to what goals were expected and the role of the GP was clear, deploying their skills and resources to offer options for the patient to consider in helping them achieve their goal. However, some patients presented with goals which GPs did not immediately align with, potentially compromised the clinical management of other chronic conditions or ones where they struggled to identify a clear mechanism to enable the goal to be achieved. At these points, a clear interactional tension could be seen to emerge, with GPs appearing to be at a crossroads where they needed to decide whether to uphold the principle of goal-setting as being patient-led or to present an alternative goal to the patient. GPs typically took the latter route, substituting mechanisms (e.g. referral to other healthcare professional) of action for the goal itself.

Similarly, where patients presented with priorities (e.g. staying well) rather than specific goals, the interaction arguably reached a state of flux with the GP needing to decide whether to continue with the goal-setting agenda, focused on how to tackle problems for patients with multi-morbidity, or to allow the possibility of no goal being set at all, with the endpoint of the discussion just being the opportunity for the patient to articulate what was troubling them. Taking the latter route would have required the GP to make a significant shift away from their institutionalised role as guide to the consultation process as well as needing him/her to step into the patient's lifeworld framing of their problem. The notion of power and patient-centredness is therefore problematised within this scenario, with a tension between how power needs to be deployed to justify the patient's and GP's time for this encounter and the need to produce a goal that is meaningful to the patient.

The different GP and patient framings of goals had consequences for how the talk was distributed, providing findings which contribute to debates about what asymmetrical interactional patterns signify about GP/patient power relations and notions of patient-centredness. Where patients and GPs displayed a shared framing of the consultation, and patients presented with goals with a clear role for the GP to support, then interactions displayed elements of symmetry. In such cases, both GP and patient could be seen to provide marked acknowledgements of the other's position to allow the interaction to proceed relatively smoothly to a point where they specified which and how goals will be achieved in six months. However, even in cases where patient and GP positions were aligned, we observed patients upholding asymmetrical relations between their own understanding of their problem and the GP position as expert. This lends further support for Pilnick and Dingwall's (2011) contention that the persistence of asymmetry is a manifestation of the function of medicine in society and not necessarily a signifier of oppressive relations. In addition, our data did display examples of asymmetry where GPs attempted to insist on a biomedical framing of the patient's problem, functioning to marginalise the person's lifeworld perspective. However, these instances were not uniform throughout consultations, rather patients and doctors could be seen to exercise power in a subtle interactional tug of war. Patients shifted from forms of passive to active resistance and back to passive resistance, and GPs could be seen asserting a biomedical view, retreating and then re-imposing their authority as the adjudicator of the patient's care. In the face of patient resistance, GPs could also be seen extending their recommendations in the pursuit of patient acceptance as the normative position of clinical consultations.

Our analysis of framings demonstrates that introducing goal-setting as a novel set of activities implicates different rules of engagement, which will not be explicitly communicated between clinician and patient. Within our data an implicit rule that empirically emerged in some consultations was that determining a strategy to achieve a patient's goals was contingent on the goal being acceptable within a biomedical framing of how to treat particular conditions. Depending on the nature of the patient goal, the GP or patient may activate this rule (e.g. by checking test results) and without explicit negotiation and agreement the grounds are set for interactional difficulties and patient resistance to occur. These actions reveal “disruptions” (Hawe, 2009) to conventional doctor-patient relations, exposing wider tensions inherent for doctors when faced with a patient's goals that are at odds with current evidence, accepted medical practice or the GP's previous experience, and a much wider ethical debate about the application of population based evidence to treat individual patients. Underpinning these disruptions lies a dichotomy implicit to goal-setting and the ideals of patient-centred care. In offering individuals the opportunity to choose goals, a distinct activity focused on values is created which precedes and is separated out from the practice of providing care. This creates an artificial linearity and doctors and patients are then required to engage in a difficult task of reintegrating those values back into a discussion of what treatments are required to tackle different chronic conditions. As Mol argues “the logic of choice tries to separate facts from values, while the logic of care attends to them jointly.” (Mol, 2008, p.46) In shining a light on goal-setting as a novel interactional activity, we have illustrated these interactional and ethical complexities surrounding doctors and their patients in the pursuit of patient-centred care.

### 4.1 Strengths, limitations and conclusions

This study was limited by the specific geographical location, the number of GP practices and the patient population. This has implications for whether we would have observed similar interactional patterns with patients with diverse chronic conditions and socio-economic circumstances, particularly for populations who have reported feelings of stigma and marginalisation as limiting their agency and ability to achieve their goals (Franklin et al., 2019b). However, whilst we were not able to establish relationships between individual characteristics and interactional style, the analysis enabled us to demonstrate a number of communication challenges for translating the broader principles of goal-setting into the reality of a doctor-patient consultation, with important implications for the training of GPs and for supporting patients to action their goals. GPs need to consider the importance of how to prepare, explain and structure the consultations, requiring a different approach where GP treatment recommendations are the normative position. Patients with long-term conditions may be encouraged to identify priorities that are important to them prior to sharing within a doctor-patient consultation.

How patients and GPs framed the goal-setting consultations in this study, and the subsequent interactional patterns we observed, were inevitably influenced by being video-recorded, although a review of video-based research suggests the impact of video does not affect study validity (Parry et al., 2016). Similarly, being asked to deliver goal-setting as part of a research project and to arrive at the consultation with three prepared goals does not reflect everyday general practice. Both GPs and patients may have been unfamiliar with participating in research, and mismatches in framings about what research activities involve may have led to some of the interactional patterns we observed. However, this research took place within an institutional context with long-established conventions about the function of medicine to treat patients and the activities that entails. As we have argued, the goal-setting consultation necessitates a shift away from these conventions, most notably taking a pro-active rather than reactive approach. In addition, patients were asked to specify goals that were meaningful to them, grounded in the contextual realities of their everyday life. In carrying out goal-setting as research participants, doctors and patients were not divorced from these histories and conventions, and our observations were primarily the result of an intersection between medicine and the lifeworld of the patient as they performed goal-setting as a novel activity.

Our findings contribute to knowledge of how the distribution of power within goal-setting consultations may involve complex shifts as interactions unfold. The goal that is finally set can be seen as a result of a negotiation of where power resides and of how to frame the patient's priorities. Enacting these priorities in terms of goals is clearly key to this negotiation, and links to Franklin et al.'s (2019a) insight into the importance of understanding how patients are predisposed towards goal-setting. Our study complements this work by suggesting that those patients who are less equipped to act as active agents of their care are also less likely to set goals that are meaningful to them in everyday life.

At the heart of these concerns lies dilemmas for notions of goal-setting and patient-centred care more generally. At what point do GPs desist from pursuing patients' goals or priorities, what is the value of doctors simply ‘bearing witness’ to patients' concerns without recommending any particular course of action, and how compatible are these concerns with finding the best and most cost-effective way to deploy the skills and knowledge of GPs to treat patients with multiple, long term conditions?

## Author contribution

**Nick Steel:** Ideas; formulation or evolution of overarching research goals and aims. Conceptualisation of overarching feasibility study. Supervision – oversight and leadership responsibility for research delivery. Formal analysis of data. Writing – review and editing. **Jamie Murdoch**: Conceptualisation of methodology and analytical approach reported in article. Writing – original draft preparation. Formal analysis of qualitative data. **Charlotte Salter**: Ideas; formulation or evolution of overarching research goals and aims. Methodology – creation of models. Conceptualisation of analysis. Formal analysis of data. Writing – review and editing. **Liz Lenaghan**: Data curation – management activities to produce and maintain research data. Investigation – conducting the research and data collection. Validation of data. Formal analysis of data. Writing – review and editing. Project administration. **Alice Shiner**: Ideas; formulation or evolution of overarching research goals and aims. Formal analysis of data. Writing – review and editing. Methodology – creation of models. **John Ford**: Ideas; formulation or evolution of overarching research goals and aims. Writing – review and editing. Methodology – creation of models. Formal analysis of data.
